# Fat-Bone Relationship in Chronic Kidney Disease—Mineral Bone Disorders: Adiponectin Is Associated with Skeletal Events among Hemodialysis Patients

**DOI:** 10.3390/diagnostics11071254

**Published:** 2021-07-13

**Authors:** Po-Cheng Chen, Shu-Wei Chang, Chih-Yu Hsieh, Jian-Chiun Liou, Jia-Feng Chang, Ting-Ming Wang

**Affiliations:** 1 Department of Urology, En Chu Kong Hospital, New Taipei City 237, Taiwan; b90401049@ntu.edu.tw; 2 Department of Civil Engineering, National Taiwan University, Taipei 106, Taiwan; changsw@ntu.edu.tw; 3 School of Biomedical Engineering, Taipei Medical University, Taipei 110, Taiwan; fish37435@hotmail.com (C.-Y.H.); jcliou@tmu.edu.tw (J.-C.L.); 4 Department of Internal Medicine, Division of Nephrology, Shuang Ho Hospital, Taipei Medical University, New Taipei City 235, Taiwan; 5 Department of Internal Medicine, Division of Nephrology, En Chu Kong Hospital, New Taipei City 237, Taiwan; 6 Renal Care Joint Foundation, New Taipei City 220, Taiwan; 7 Department of Nursing, Yuanpei University of Medical Technology, Hsinchu 300, Taiwan; 8 Department of Orthopaedic Surgery, School of Medicine, National Taiwan University, Taipei 106, Taiwan; dtorth76@yahoo.com.tw; 9 Department of Orthopaedic Surgery, National Taiwan University Hospital, Taipei 106, Taiwan

**Keywords:** adiponectin, bone fracture, chronic kidney disease, mineral disorder, hemodialysis

## Abstract

Background: The risk of skeletal events is rising in parallel with the burden of chronic kidney disease and mineral bone disorder (CKD-MBD), whilst the role of the fat-bone axis in CKD-MBD remains elusive. Adiponectin derived from adipocytes has emerged as a valid biomarker of low bone mineral density and increased marrow adiposity. We aimed to explore the association between adiponectin and bone fracture (BF) risks in patients with maintenance hemodialysis (MHD). Methods: Serum concentrations of adiponectin and bio-clinical data were determined at study entry. The Cox proportional hazard regression analyses were used to assess unadjusted and adjusted hazard ratios (aHRs) of adiponectin and various clinical predictors for BF risks. The predictive accuracy of adiponectin for BF events was evaluated by receiver operating characteristic (ROC) curve analysis. Results: Age and serum concentrations of adiponectin, phosphate, and intact parathyroid hormone were significantly associated with higher risks of BF. With respect to the risk of BF events, the cumulative event-free survival curves differed significantly between the high and low concentration groups of adiponectin (*p* = 0.02). In multivariable analysis, higher adiponectin levels were associated with an incremental risk of BF (adjusted hazard ratios (aHRs): 1.08 (95% confidence interval (CI): 1.01–1.15, *p* < 0.05). The ROC analysis of adiponectin cutoff point concentration (18.15 ug/mL) for prediction of BF showed 0.66 (95% CI = 0.49 to 0.84). Conclusion: Adiponectin was associated with an incremental risk of BF that could serve as a potential predictor of BF in MHD patients. In the high-risk population with hyperphosphatemia, an elevated adiponectin level could alert clinicians to the urgent need to correct mineral dysregulation and undertake further bone survey.

## 1. Introduction

Patients with chronic kidney disease (CKD) have an exceedingly high risk of skeletal events, and those receiving maintenance hemodialysis (MHD) top the list of bone fracture (BF) cases [1]. CKD related mineral bone disorders (MBD) are intricately involved in cardiovascular diseases and the development of all-cause mortality in MHD patients [2,3]. When it comes to the crosstalk between fat and bone, both experimental research and observational data indicate a negative correlation between fat mass and bone mass, suggesting that fat mass may interact with bone mass. [4,5]. Meanwhile, bone marrow adiposity tracks with obesity, and vice versa [6,7]. Intriguingly, osteoblasts (build new bone) and adipocytes derived from the same mesenchymal progenitor [8]. A myriad of studies indicate that circulating levels of adiponectin derived from adipocytes are inversely associated with bone mineral density (BMD) in the general population [9]. Furthermore, adipocytes in the bone marrow have long been recognized as “passive filler”, and when BMD is low, adiponectin is positively correlated with bone marrow adiposity [10]. Emergent evidence reveals adipokines play a central role in the reciprocal regulation of mesenchymal stem cell (MSC) differentiation into osteoblasts or adipocytes [11]. Notably, adiponectin concentrations (in the range of µg/mL) in the circulation are much higher than other similar hormones and cytokines [3]. In light of this, adiponectin may act as a critical mediator in the fat-bone axis. Indeed, analysis of the association between adiponectin levels and BF risk in the largest prospective cohort study showed that men with the highest adiponectin tertile had a 94% incremental BF risk compared with the lowest tertile [12]. Likewise, adiponectin is considered as an independent predictor of osteoporotic BF at different bone sites in another large observational study of Asian postmenopausal women [13]. Despite previous documented implications, the predictive value of adiponectin for BF in patients with CKD-MBD remains elusive. Accordingly, we aim to explore the association between adiponectin and BF risks in a MHD population.

## 2. Methods

### 2.1. Participants in the Cohort

The study was approved by the Research Ethics Review Committee of En Chu Kong Hospital (ECKIRB1070102) in accordance with the ethical standards of the committee and the Helsinki declaration for research in humans. Written informed consent was obtained from the participants of this study. The relevant details of the research methods were described previously [1,2]. Patients undergoing MHD treatment for at least three months were eligible for enrollment. All patients had to be older than 18 years of age and receive thrice-weekly hemodialysis, and 106 MHD patients, willing to participate in the study, were enrolled. Twenty patients were excluded from the study because of loss of follow up, death prior to BF events, inadequate dialysis, terminal illness, active infections, advanced cancer, active hepatitis, severe protein-energy wasting, or incomplete data.

### 2.2. Assessment of Exposures

Circulating adiponectin levels and bio-clinical parameters were ascertained at baseline. Serum concentrations of adiponectin were measured by a commercial quantitative enzyme-linked immunosorbent (ELISA) assay (Human adiponectin ELISA Kit (R&D Systems, Minneapolis, MN, USA) in accordance with the manufacturer’s instructions. The study subjects were categorized into low-level and high-level groups by mean value of serum adiponectin concentration (11.15 μg/mL). The following bio-demographic and laboratory parameters of each patient were recorded at baseline: age, gender, hypertension, DM (diabetes mellitus), HD vintage (length of time on dialysis), pre-dialysis blood urea nitrogen, Kt/V urea (solute removal during dialysis), normalized protein catabolic rate (nPCR), creatinine, potassium, calcium, phosphorus, non-hepatic alkaline phosphatase (NHALP), alanine aminotransferase, aspartate aminotransferase, albumin, uric acid, total cholesterol, triglyceride, hemoglobin, hematocrit, calcium-phosphate product, and intact parathyroid hormone (iPTH). Patients with hepatobiliary diseases were excluded in our study after careful chart review. Thus, the origins of elevated alkaline phosphatase (ALP) concentrations were determined to be non-hepatic, such as CKD-mineral bone diseases or skeletal events [14]. NHALP is one of the most important osteoblast marker proteins for bone mineralization, hydrolysis of mineralization inhibitor pyrophosphate, and extra-osseous vascular calcification associated mortality in CKD patients [15]. The HD vintage was defined as the duration of time between the first day of HD treatment and the first day that the patient entered the cohort. Pre-dialysis blood samples were obtained from the existing vascular access for further analyses. We adjusted serum calcium according to the following equation: adjusted calcium = measured calcium + ((4.0-serum albumin in g/dL) × 0.8). All laboratory tests were performed by the standard procedures with certified methods. The estimated glomerular filtration rate (eGFR) was calculated using the CKD-Epidemiology Collaboration formula. The Ministry of Health and Welfare of Taiwan defines normal body mass index (BMI): 18.5 to <24 kg/m^2^; obesity: BMI ≥ 27 kg/m^2^; overweight: BMI 24 to <27 kg/m^2^; and underweight: BMI < 18.5 kg/m^2^.

### 2.3. Ascertainment of Outcomes

The primary outcome was the incidence of BF events, which was defined as a new diagnosis of BF for any sites that occurred during follow-up, including skull, vertebrae, ribs, shoulder, humerus, forearm, wrist, hip, pelvis, femur, knee, leg, ankle, fingers, toes, or other skeletons. The occurrence of BF events was evaluated by clinical diagnosis (either from inpatient chart review or outpatient medical records) with evidence of BFs in the formal image report (computed tomography or roentgenogram) by radiologists. Vertebral fractures were assessed with Genant’s method as described previously [14]. The outcome information was centrally assessed by trained clinicians, nephrologists, and radiologists.

### 2.4. Statistical Analysis

Continuous variables were presented as mean ± standard deviation, and the categorical variables were expressed as number (%). Correlation coefficients between covariates of interest were calculated. Univariate Cox regression analysis was performed to investigate the independence of risk factors associated with BF. Unadjusted and multivariable adjusted hazard ratios (aHRs) of BF risks were calculated for serum adiponectin concentrations in the Cox regression model. The cumulative survival probability and proportional hazards were categorized according to the higher and lower concentration groups of adiponectin with graphical methods. To assess the predictive accuracy of circulating adiponectin levels for mortality, the area under the ROC curve (AUC) was used as the criterion in accordance with our previous research [2,16]. An AUC of 0.5 indicates no predictive ability, whereas a value of 1 represents perfect predictive ability. A *p*-value < 0.05 was considered statistically significant. We used the PASW Statistics SPSS version 22.0 (IBM, Armonk, NY, USA) to analyze all bio-clinical data of MHD patients.

## 3. Results

### 3.1. Adiponectin, NHALP, Phosphate, Calcium-Phosphate Product, and iPTH Levels Were Significantly Different between BF Event and Event-Free Groups

Baseline bio-clinical data of the whole study population with comparisons between BF and BF-free survivors are summarized in Table 1. The final study sample included 86 MHD patients with complete medical records and follow-up. The mean duration of follow-up was 23.9 ± 12.7 months. The overall incidence rate of BFs was 15.1% over 2058 person-months of follow-up, corresponding to an annual event rate of 7.6%. The mean age of whole study patients was 62.6 ± 10.3 years. The mean BMI was 23.7 ± 2.9 kg/m^2^ (male:female = 39:47); approximately 12.8% of the study patients had obesity (male:female = 7:4), 27.9 % had overweight (male:female = 12:12) and 3.5 % had underweight (male:female = 0:3). The BF event group compared with the BF-free group had a lower level of body weight (58.0 ± 7.3 versus 60.5 ± 10.0 kg), suggesting loss of muscle mass (sarcopenia) is related to raised adiponectin levels and unfavorable outcomes. However, the statistical result was insignificant (*p*-value = 0.27). The mean concentration of adiponectin was 11.2 ± 7.8 μg/mL. Adiponectin, NHALP, phosphate, calcium-phosphate product, and iPTH levels were significantly different between the event and event-free group. Specifically, our data demonstrated adiponectin (17.3 ± 13.6 vs. 10.1 ± 5.8 μg/mL), NHALP (111.9 ± 92.7 vs. 70.7 ± 26.3 IU/L), phosphate (5.9 ± 1.6 vs. 4.1 ± 1.3 mg/dL), calcium-phosphate product (51.5 ± 15.9 vs. 38.3 ± 12.7) and iPTH (367.8 ± 359.2 vs. 133.5 ± 127.6 pg/mL), respectively. Compared with the non-BF group, the mean age of the BF group was older (61.8 ± 10.4 vs. 66.8 ± 8.7, respectively). However, the *p*-value was 0.11. The BF group compared with the BF-free group favored females (76.9% vs. 50.7%, *p* = 0.08) and had higher prevalence of DM (61.5% vs. 39.7%, *p* = 0.15), lower levels of serum calcium (9.1 ± 0.7 vs. 9.3 ± 0.8 mg/dL, *p* = 0.49), hemoglobin (10.1 ± 1.4 vs. 10.8 ± 1.4 g/dL, *p* = 0.11) and hematocrit (30.6 ± 4.2 vs. 32.3 ± 4.2%, *p* = 0.19), respectively.

All patients were further stratified by the mean value of adiponectin concentration (11.2 ± 7.8 μg/mL) into two categories: the high concentration group (>11.15 μg/mL) and the low concentration group (<11.15 μg/mL). Table 2 summarizes the comparison of BF events and relevant bio-clinical data between the high and low concentration groups according to the mean value of adiponectin. The total BF events in the high concentration and low concentration groups were 8 and 5, corresponding to an annual BF rate of 12.9% and 4.6%, respectively. The total incidence rate of BF events (25.8% and 9.1%, respectively), annual BF incidence rates (12.9% and 4.6%, respectively), adiponectin (18.9 ± 8.2μg/mL, 6.8 ± 2.5 μg/mL, respectively), triglyceride (156.1 ± 121.1 mg/dL and 238.8 ± 202.9 mg/dL, respectively), and platelet levels (175.9 ± 55.4 k/μL, and 207.0 ± 68.3 k/μL, respectively) were significantly different between adiponectin concentration groups. Although patients in the high concentration group had higher serum levels of phosphate and lower levels of blood glucose and calcium, the results for statistical difference were insignificant between groups (*p*-value = 0.23, 0.09, and 0.21, respectively).

### 3.2. The Higher Concentration Group of Adiponectin Was Associated with an Incremental Risk of BF Events

**Figure 1:**
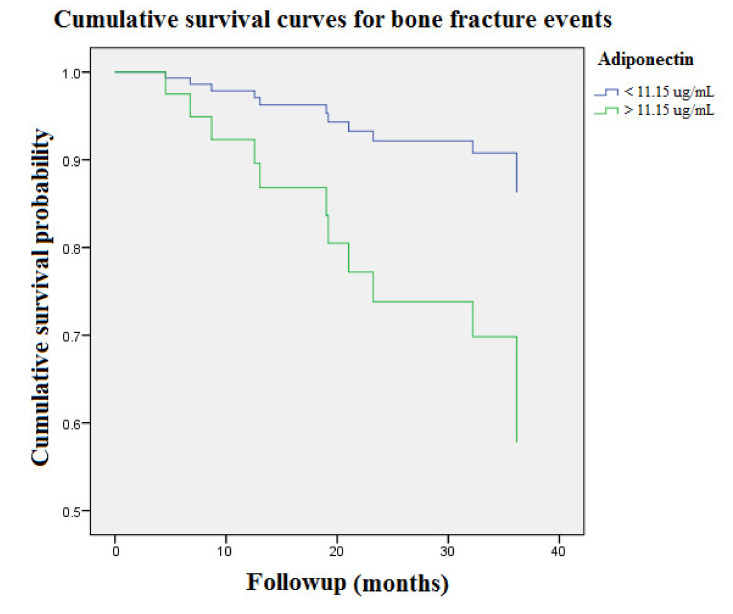
Cumulative event-free survival curves of bone fracture risks among patients with respect to the higher and lower concentration groups of adiponectin over 2058 person-months of follow-up. The blue line denotes patients with adiponectin concentration < 11.15 ug/mL. The green line denotes patients with adiponectin concentration > 11.15 ug/mL.

Figure 1 illustrates cumulative event-free survival curves of BF events according to higher and lower concentration groups of adiponectin (>11.15 and <11.15 ug/mL, respectively) over 2058 person-months of follow-up. With respect to the lower concentration group of adiponectin (<11.15 ug/mL) as reference, the patients in the higher concentration group (>11.15 ug/mL) were associated with an incremental risk of BF events (aHR: 3.7 [95% CI: 1.2–11.4], *p* = 0.02).

### 3.3. In Multivariate Cox Regression Analysis, the Associations between the Highest Adiponectin Tertile and Risks of BF Events Remain Robust

Table 3 summarizes the clinical prognostic factors of BF events from the unadjusted univariate and adjusted multivariate Cox regression model in predicting the occurrence of BF events. The univariate Cox regression analysis without adjustment (Model 1) demonstrated the high concentration group (adiponectin > 11.2 ug/mL) was independently associated with the risk of BF events (aHR: 1.05 [95% CI: 1.01–1.09]). Furthermore, age (aHR: 1.09 [95% CI: 1.02–1.17]), NHALP (aHR: 1.01 [95% CI: 1.00–1.02]), phosphate (aHR: 1.73 [95% CI: 1.33–2.25]), and iPTH (aHR: 1.03 [95% CI: 1.01–1.04]) were all independent risk factors of BF events in the current study. The multivariate Cox regression model (Model 2) adjusted for all independent risk factors of BFs demonstrated the high adiponectin group was associated with BF risks [aHR: 1.08 (95% CI: 1.01–1.15)]. The following independent risk factors of BF events remained robust after multivariate adjustment: age (aHR: 1.20 [95% CI: 1.08–1.35]), and phosphate (aHR: 1.82 [95% CI: 1.21–2.72]), respectively. The circulating levele of iPTH showed a trend that was associated with the risk of BF events after multivariate adjustment, whereas the *p*-value was 0.11.

### 3.4. ROC Curve Analysis of Serum Adiponectin Cutoff Point Concentration (18.15 ug/mL) for Prediction of BF Events

**Figure 2:**
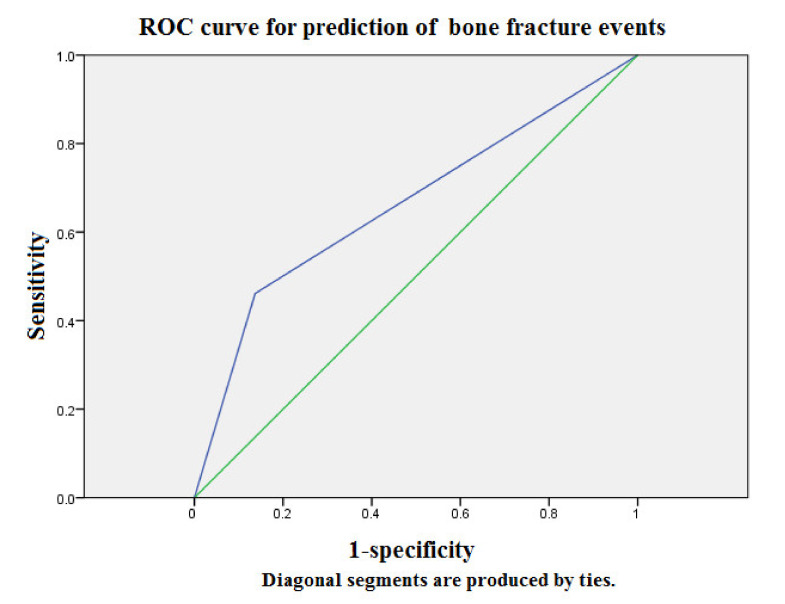
The ROC analysis of adiponectin concentration for prediction of BF events showed acceptable discriminatory power in the whole study population over 30 months of follow-up. The AUC provided by adiponectin tertiles for all-cause mortality was 0.66 (95% CI = 0.49 to 0.84). Source of the curve: green line = adiponectin concentrations; blue line = reference. AUC = area under curve; ROC = receiver operating characteristics.

Figure 2 displays the ROC analysis of adiponectin concentrations for prediction of BF events in the whole study population over 2058 person-months of follow-up. The AUC provided by the adiponectin concentrations for BF events is 0.66 (95% CI = 0.49 to 0.84), and the cutoff point concentration was 18.15 ug/mL. Collectively, the ROC analysis of adiponectin concentration for prediction of BF events showed acceptable discriminatory power. In light of underdiagnosis of bone loss in CKD-MBD, an elevated circulating level of adiponectin serves as an early warning sign for timely correction of mineral dysregulation and appropriate bone survey to prevent BF events in the high risk MHD population.

## 4. Discussion

High morbidity and mortality rates correlate tightly with progressive CKD-MBD that are intricately involved in underlying metabolic bone alterations and bone marrow microenvironment (e.g., adipocytes, mesenchymal stem cells, osteoblasts and osteoclasts) [10,17,18,19,20]. Adipocytes in bone marrow, or termed marrow adipose tissue, reside in the bone microenvironment in close contact to all types of bone cells. From bedside to bench, the relationship between the fat-bone axis and osteoporotic BF events is evident [10,17,18,19,20]. Despite these molecular connections between fat and bone, none of the prior research investigated the association between CKD-MBD and the fat-bone axis. Therefore, we introduce a novel diagnostic predictor by conducting a comprehensive evaluation of adipocytes-derived adiponectin and dysregulated mineral metabolism for BF events in MHD patients. Specifically, higher circulating levels of adiponectin are independently related to BF events after adjusting for traditional risk factors of CKD-MBD, including phosphate, iPTH and NHALP. Several important findings in this work deserve further discussion.

Clinical observational studies report an incremental increase in BF incidence from 15.0 to 20.5, 24.2, 31.2, and 46.3/1000 person-years for CKD stages 1 to 2, 3a, 3b, and 4, respectively [21]. The associated BF risk reaches nearly five times higher in CKD patients with stage 5 versus stage 1–2. In concordance with the prior research, our data demonstrated the overall incidence rate of BFs was 15.1% in the HD population, corresponding to an annual event rate of 7.6% (Table 1). In our cohort study, adiponectin, NHALP, phosphate, calcium-phosphate product and iPTH were significantly different between BF event and event-free groups. BF patients had a trend to age older, favor females and manifest higher prevalence of DM, lower levels of hemoglobin and hematocrit (*p*-value = 0.11, 0.08, 0.15, 0.11 and 0.19, respectively).). Because our study population were hemodialysis patients, the overall value of eGFR was extremely low (4.2 ± 1.2 mL/min). The BF event group compared with the BF-free group had a lower level of eGFR (3.4 ± 0.6 versus 4.4 ± 1.2 mL/min), suggesting a potential association between eGFR and bone events. However, there is only a trend toward statistical significance (*p*-value = 0.07). In the subgroup analysis of male patients (*n* = 39), the eGFR is inversely and positively associated with BF events and adiponectin levels (the correlation coefficient = −0.16 and 0.23, respectively). Likewise, the statistical result was insignificant (*p*-value = 0.34 and 0.16). After dividing into two groups according to the mean value of circulating adiponectin (11.2 ug/mL), the annual BF rates significantly differed between the high-level and low-level groups (12.9% and 4.6%, respectively) (Table 2). Furthermore, we examined the associations between adiponectin and other clinical risk factors for BF events in the Cox proportional hazard regression model (Table 3). After multivariate adjustment for age, NHALP, phosphate and iPTH, the associations between adiponectin and risks of BF events remained robust (*p* < 0.05). During 2058 person-months of follow-up, the patients in the higher concentration group (>11.15 ug/mL) had an associated incremental risk of BF events with respect to the lower concentration group of adiponectin (<11.15 ug/mL) as reference (*p* < 0.05). (Figure 1). Notably, the ROC analysis demonstrated the AUC provided by the adiponectin concentrations for prediction of BF events is 0.66 (95% CI = 0.49 to 0.84), and the cutoff point concentration was 18.15 ug/mL, suggesting that adiponectin could serve as a potential predictor of BF events (Figure 2). Naot et al. reported serum adiponectin concentration is a potent prognostic parameter of associated BMD loss and osteoporotic BF risks from a number of large population studies [9]. Considering BMD measurement is inaccessible to routine clinical practice, elevated circulating levels of adiponectin could provide an alternate surrogate marker of low BMD and potential BF events in the high risk MHD population with mineral dysregulation.

A new nomenclature of CKD-related bone fragility termed uremic osteoporosis is a typical manifestation of CKD-induced bone material abnormalities, particularly in bone elasticity [22]. Uremic osteoporosis is a unique concept describing bone illness in CKD-MBD population, and the pathophysiological background is not clearly illustrated. Moreover, uremic osteoporosis is similar but somehow different from conventional skeletal osteoporosis in general population. For instance, the reduction of BMD occurred mainly in the hip instead of the spine, and was associated with age and secondary hyperparathyroidism in CKD patients [23]. Beyond the risk factor of age, the evaluation of BF risks was not simply the same as the general population due to high prevalence of multiple comorbidity and altered microenvironment for bone in CKD-MBD, e.g., uremic burden, hyperphosphatemia, hypocalcemia, secondary hyperparathyroidism, hypovitaminosis D, anemia, and protein-energy wasting, etc. [24]. Indeed, conventional risk factors for BFs, such as gender and DM, did not show significant results in the univariate Cox regression model. Intriguingly, adiponectin seems to be a key player in crosstalk between the fat-bone axis and CKD-MBD. In fact, adipokines regulate bone mass in multiple ways [11]. On one hand, marrow adipocytes can interact with osteoblasts and their precursors in a paracrine loop. In experimental research, coculture with adipocyte inhibited MC3T3-E1 osteoblasts with an increased adipogenesis and decreased osteoblastogenesis [25]. In addition, peroxisome proliferator-activated receptor γ (PPARγ) activation selectively promotes adipogenesis from mesenchymal stem cells, leading to the accumulation of adipocytes with a concomitant reduction of osteoblast numbers in bone marrow [26]. The above findings suggest that PPARγ activation commits mesenchymal stem cells to differentiate into adipocytes, away from the osteoblastic lineage. On the other hand, the adipokine leptin could modulate bone mass and body weight through the central nervous system [11]. Considering the bone effects of adipokines, we are conducting an observational study for fatal or non-fatal events, circulating levels of adipokines and other novel biomarkers to prove that MHD patients suffer high risk of adverse clinical outcomes subsequent to BF events (unpublished data). In the United States, the medical expenditure for BFs is more than $1 billion annually [14]. To reduce medical expenditures, the assessment of BF risks and prevention strategies should be considered in nephrology practice.

We acknowledged several limitations about this study. To begin with, our cohort size was relatively small and constituted only an Asian population, and the results should be interpreted with caution. Further international studies with larger sample sizes and longer follow-up periods are required to confirm the findings of this preliminary study. Next, the circulating biochemical values were obtained at a single time point upon study entry, and variability over time might not be reflected. In addition, the data of BMD monitored by dual-energy x-ray absorptiometry or other bone quality measurements were not performed in our patients. Thus the severity of osteoporosis was not accessible.

## 5. Conclusions

Since the risk stratification can assist clinicians with an appropriate diagnostic test and corresponding therapeutic strategy in advance, searching for a practical biomarker-based BF predictor is of prime importance for MHD patients with CKD-MBD. Considering the inaccessibility of BMD measurement in routine clinical practice, elevated circulating levels of adiponectin could serve as an alternate surrogate marker of low BMD and BF events in the CKD population with mineral dysregulation. In the high-risk population with hyperphosphatemia, an elevated circulating level of adiponectin could alert clinicians to the urgent need to correct mineral dysregulation and conduct further bone surveys.

## Figures and Tables

**Table 1:** Baseline bio-clinical data of the whole study population with comparisons between the bone fracture group and fracture-free survivors.

	Overall Population (*n* = 86)	BF Events (*n* = 13)	Event-Free Survivors (*n* = 73)
Age (years)	62.6 ± 10.3	66.8 ± 8.7	61.8 ± 10.4
Male, *n* (%)	39 (45.3)	3 (23.1)	36 (49.3)
Female, *n* (%)	47 (54.7)	10 (76.9)	37 (50.7)
Body weight (kg)	60.1 ± 9.6	58.0 ± 7.3	60.5 ± 10.0
Body mass index (kg/m^2^)	23.7 ± 2.9	23.7 ± 3.1	23.7 ± 2.9
Diabetes mellitus, *n* (%)	37 (43.0)	8 (61.5)	29 (39.7)
Hypertension, *n* (%)	34 (39.5)	4 (30.8)	30 (41.1)
Hemodialysis vintage (months)	32.1 ± 26.7	36.8 ± 22.5	31.3 ± 27.4
Kt/V urea	1.6 ± 0.3	1.5 ± 0.3	1.6 ± 0.3
**Adiponectin (μg/mL)**	**11.2 ± 7.8**	**17.3 ± 13.6**	**10.1 ± 5.8**
**NHALP (IU/L)**	**76.9 ± 45.0**	**111.9 ± 92.7**	**70.7 ± 26.3**
nPCR	1.1 ± 0.3	1.0 ± 0.2	1.1 ± 0.2
Albumin (g/dL)	3.9 ± 0.5	3.9 ± 0.2	3.9 ± 0.5
Aspartate aminotransferase (IU/L)	16.0 ± 7.0	14.8 ± 3.6	16.2 ± 7.4
Alanine aminotransferase (IU/L)	13.5 ± 10.2	11.5 ± 6.5	13.9 ± 10.7
Total cholesterol (mg/dL)	192.5 ± 50.8	204.0 ± 42.5	190.5 ± 52.1
Triglyceride (mg/dL)	209.0 ± 181.4	228.3 ± 224.5	205.6 ± 174.3
Blood glucose (mg/dL)	143.2 ± 77.1	155.9 ± 100.9	140.9 ± 72.7
Blood urea nitrogen (mg/dL)	65.5 ± 16.0	72.6 ± 18.8	64.3 ± 15.3
Creatinine (mg/dL)	10.3 ± 1.7	11.2 ± 1.0	10.2 ± 1.7
eGFR (ml/min)	4.2 ± 1.2	3.4 ± 0.6	4.4 ± 1.2
Uric acid (mg/dL)	7.5 ± 1.3	7.6 ± 1.3	7.4 ± 1.3
Potassium (mmol L^−1^)	4.6 ± 0.8	4.8 ± 0.8	4.5 ± 0.8
Calcium (mg/dL)	9.3 ± 0.7	9.1 ± 0.7	9.3 ± 0.8
**Phosphate (mg/dL)**	**4.4 ± 1.5**	**5.9 ± 1.6**	**4.1 ± 1.3**
**Calcium-phosphate product**	**40.3 ± 14.0**	**51.5 ± 15.9**	**38.3 ± 12.7**
**iPTH (pg/mL)**	**168.9 ± 197.9**	**367.8 ± 359.2**	**133.5 ± 127.6**
Hemoglobin (g/dL)	10.7 ± 1.4	10.1 ± 1.4	10.8 ± 1.4
Hematocrit (%)	32.0 ± 4.2	30.6 ± 4.2	32.3 ± 4.2
Platelet (k/μL)	195.8 ± 65.4	197.2 ± 36.8	195.6 ± 69.4

Continuous variables were expressed as mean ± SD. Categorical variables are expressed as n (%). Boldface indicates where the values differ significantly between event-free survivors and non-survivors. eGFR = estimated glomerular filtration rate; iPTH = intact parathyroid hormone; Kt/V urea = dialysis dose calculated by Gotch’s method; NHALP = non-hepatic alkaline phosphatase; nPCR = normalized protein catabolic rate; adiponectin = p-cresyl sulfate. BMI is defined as the weight in kilograms divided by the square of the height in metres (kg/m^2^). The eGFR was calculated using Chronic Kidney Disease-Epidemiology Collaboration formula.3.2. BF event rates, serum concentrations of platelet and triglyceride were significantly different between the higher and lower concentration groups of adiponectin.

**Table 2:** Comparison of BF events and bio-clinical parameters between higher and lower concentration groups according to the mean value of adiponectin.

	High Concentration Group > 11.15 μg/mL	Low Concentration Group < 11.15 μg/mL	*p*-Value
Patients, *n* (%)	31 (36.0)	55 (64.0)	
**BF events, *n* (%)**	**8 (** **25.8)**	**5 (** **9.1)**	**<0.05**
**Annual BF rates (%)**	**12.9**	**4.6**	**<0.05**
Age (years)	62.3 ± 10.4	62.7 ± 10.3	0.82
Male, *n* (%)	13 (41.9)	26 (47.3)	0.64
Diabetes mellitus, *n* (%)	13 (41.9)	24 (43.6)	0.88
Hypertension, *n* (%)	14 (45.2)	20 (36.4)	0.43
Hemodialysis vintage (months)	36.1 ± 26.5	29.9 ± 26.8	0.31
**Adiponectin (μg/mL)**	**18.9 ± 8.2**	**6.8 ± 2.5**	**<0.01**
Kt/V urea	1.6 ± 0.3	1.6 ± 0.3	0.79
NHALP	77.1 ± 60.3	76.8 ± 34.1	0.98
nPCR (g/kg/day)	1.1 ± 0.3	1.1 ± 0.3	0.99
Albumin (g/dL)	3.8 ± 0.5	3.9 ± 0.4	0.53
Blood urea nitrogen (mg/dL)	66.5 ± 17.0	65.0 ± 15.6	0.68
Total cholesterol (mg/dL)	190.2 ± 45.8	193.8 ± 53.7	0.76
**Triglyceride (mg/dL)**	**156.1 ± 121.1**	**238.8 ± 202.9**	**<0.05**
Blood glucose (mg/dL)	124.2 ± 69.6	153.9 ± 79.6	0.09
Uric acid (mg/dL)	7.4 ± 1.4	7.5 ± 1.2	0.88
Potassium (mmol L^−1^)	4.6 ± 0.8	4.5 ± 0.8	0.57
Calcium (mg/dL)	9.1 ± 0.7	9.3 ± 0.8	0.21
Phosphate (mg/dL)	4.7 ± 1.7	4.2 ± 1.4	0.23
Calcium-phosphate product	41.9 ± 15.7	39.3 ± 13.0	0.42
Intact parathyroid hormone (pg/mL)	186.7 ± 237.2	158.9 ± 173.4	0.54
Hemoglobin (g/dL)	10.9 ± 1.2	10.6 ± 1.6	0.30
Hematocrit (%)	32.8 ± 3.7	31.6 ± 4.5	0.24
Platelet (k/μL)	175.9 ± 55.4	207.0 ± 68.3	<0.05

Continuous variables were expressed as mean ± SD. Categorical variables are expressed as *n* (%). Boldface indicates where the values differ significantly between adiponectin tertiles. BF = bone fracture. Kt/V urea = dialysis dose calculated by Gotch’s method. nPCR = normalized protein catabolic rate.

**Table 3:** The associations between adiponectin and other clinical risk factors for bone fracture events in the Cox proportional hazard regression model.

	Model 1		Model 2	
	HR (95% CI)	*p* Valve	HR (95% CI)	*p* Valve
Age (per year increase)	1.09 (1.02–1.17)	<0.01	1.20 (1.08–1.35)	<0.01
Gender (male vs. female)	0.29 (0.08–1.04)	0.06		
Diabetes mellitus (yes vs. no)	1.96 (0.64–6.01)	0.24		
Adiponectin (per unit increase)	1.05 (1.01–1.09)	<0.01	1.08 (1.01–1.15)	<0.05
HD vintage (per month increase)	1.01 (0.99–1.03)	0.47		
Kt/V urea (per unit increase)	0.22 (0.04–1.31)	0.10		
Albumin (per unit increase)	0.54 (0.12–2.44)	0.42		
NHALP (per unit increase)	1.01 (1.00–1.02)	<0.01	1.00 (0.99–1.01)	0.67
AST (per unit increase)	0.99 (0.89–1.09)	0.81		
ALT (per unit increase)	0.96 (0.89–1.04)	0.36		
Total cholesterol (per unit increase)	1.00 (0.99–1.01)	0.86		
Triglyceride (per unit increase)	1.00 (1.00–1.00)	0.82		
Blood glucose (per unit increase)	1.00 (1.00–1.01)	0.68		
Blood urea nitrogen (per unit increase)	1.03 (1.00–1.06)	0.06		
Uric acid (per unit increase)	1.08 (0.73–1.60)	0.71		
Potassium (per unit increase)	1.48 (0.76–2.90)	0.25		
Calcium (per unit increase)	0.62 (0.27–1.45)	0.27		
Phosphate (per unit increase)	1.73 (1.33–2.25)	<0.01	1.82 (1.21–2.72)	<0.01
iPTH (per 10 unit increase)	1.03 (1.01–1.04)	<0.01	1.03 (1.00–1.06)	0.11
Hemoglobin (per unit increase)	0.69 (0.47–1.01)	0.06		
Hematocrit (per unit increase)	0.90 (0.78–1.02)	0.11		
Platelet (per unit increase)	1.00 (0.99–1.01)	0.64		

CI = confidence interval; HR = hazard ratio. Model 1: Univariate Cox proportional hazards regression model; Model 2: Multivariate Cox proportional hazards regression model: Adjusted for all independent risk factors in the model 1. ALT = Alanine aminotransferase; AST = Aspartate aminotransferase; HD = Hemodialysis; iPTH = intact parathyroid hormone; NHALP = non-hepatic alkaline phosphatase.

## Data Availability

The numeric data used to support the findings of this study are available from the corresponding author, Jia-Feng Chang, upon reasonable request. Corresponding author’s email: cjf6699@gmail.com.

## References

[1] Ko W.-C., Choy C.-S., Lin W.-N., Chang S.-W., Liou J.-C., Tung T.-H., Hsieh C.-Y., Chang J.-F. (2018). Galectin-3 Interacts with Vascular Cell Adhesion Molecule-1 to Increase Cardiovascular Mortality in Hemodialysis Patients. J. Clin. Med..

[2] Chang J.-F., Chou Y.-S., Wu C.-C., Chen P.-C., Ko W.-C., Liou J.-C., Hsieh C.-Y., Lin W.-N., Wen L.-L., Chang S.-W. (2020). A Joint Evaluation of Neurohormone Vasopressin-Neurophysin II-Copeptin and Aortic Arch Calcification on Mortality Risks in Hemodialysis Patients. Front. Med..

[3] Hung K.C., Chang J.F., Hsu Y.H., Hsieh C.Y., Wu M.S., Wu M.Y., Chiu I.J., Syu R.S., Wang T.M., Wu C.C. (2020). Therapeutic Effect of Calcimimetics on Osteoclast-Osteoblast Crosslink in Chronic Kidney Disease and Mineral Bone Disease. Int. J. Mol. Sci..

[4] Fu X., Ma X., Lu H., He W., Wang Z., Zhu S. (2011). Associations of fat mass and fat distribution with bone mineral density in pre-and postmenopausal Chinese women. Osteoporos. Int..

[5] Zhao L.-J., Liu Y.-J., Liu P.-Y., Hamilton J., Recker R.R., Deng H.-W. (2007). Relationship of obesity with osteoporosis. J. Clin. Endocrinol. Metab..

[6] Devlin M.J., Rosen C.J. (2015). The bone–fat interface: Basic and clinical implications of marrow adiposity. Lancet Diabetes Endocrinol..

[7] Schwartz A.V., Sigurdsson S., Hue T.F., Lang T.F., Harris T.B., Rosen C.J., Vittinghoff E., Siggeirsdottir K., Sigurdsson G., Oskarsdottir D. (2013). Vertebral bone marrow fat associated with lower trabecular BMD and prevalent vertebral fracture in older adults. J. Clin. Endocrinol. Metab..

[8] Zaidi M. (2007). Skeletal remodeling in health and disease. Nat. Med..

[9] Naot D., Musson D.S., Cornish J. (2017). The Activity of Adiponectin in Bone. Calcif. Tissue Int..

[10] Veldhuis-Vlug A.G., Rosen C.J. (2018). Clinical implications of bone marrow adiposity. J. Intern. Med..

[11] Zaidi M., Buettner C., Sun L., Iqbal J. (2012). Minireview: The link between fat and bone: Does mass beget mass?. Endocrinology.

[12] Barbour K.E., Zmuda J.M., Boudreau R., Strotmeyer E.S., Horwitz M.J., Evans R.W., Kanaya A.M., Harris T.B., Bauer D.C., Cauley J.A. (2011). Adipokines and the risk of fracture in older adults. J. Bone Min. Res..

[13] Nakamura Y., Nakano M., Suzuki T., Sato J., Kato H., Takahashi J., Shiraki M. (2020). Two adipocytokines, leptin and adiponectin, independently predict osteoporotic fracture risk at different bone sites in postmenopausal women. Bone.

[14] Yeh J.C., Wu C.C., Choy C.S., Chang S.W., Liou J.C., Chen K.S., Tung T.H., Lin W.N., Hsieh C.Y., Ho C.T. (2018). Non-Hepatic Alkaline Phosphatase, hs-CRP and Progression of Vertebral Fracture in Patients with Rheumatoid Arthritis: A Population-Based Longitudinal Study. J. Clin. Med..

[15] Chang J.F., Liu S.H., Lu K.C., Ka S.M., Hsieh C.Y., Ho C.T., Lin W.N., Wen L.L., Liou J.C., Chang S.W. (2020). Uremic Vascular Calcification Is Correlated With Oxidative Elastic Lamina Injury, Contractile Smooth Muscle Cell Loss, Osteogenesis, and Apoptosis: The Human Pathobiological Evidence. Front. Med..

[16] Chang J.-F., Chen P.-C., Hsieh C.-Y., Liou J.-C. (2021). A Growth Differentiation Factor 15-Based Risk Score Model to Predict Mortality in Hemodialysis Patients. Diagnostics.

[17] Pimentel A., Ureña-Torres P., Bover J., Luis Fernandez-Martín J., Cohen-Solal M. (2021). Bone Fragility Fractures in CKD Patients. Calcif. Tissue Int..

[18] Pimentel A., Bover J., Elder G., Cohen-Solal M., Ureña-Torres P.A. (2021). The Use of Imaging Techniques in Chronic Kidney Disease-Mineral and Bone Disorders (CKD-MBD)-A Systematic Review. Diagnostics.

[19] Pimentel A., Ureña-Torres P., Zillikens M.C., Bover J., Cohen-Solal M. (2017). Fractures in patients with CKD-diagnosis, treatment, and prevention: A review by members of the European Calcified Tissue Society and the European Renal Association of Nephrology Dialysis and Transplantation. Kidney Int..

[20] Tentori F., McCullough K., Kilpatrick R.D., Bradbury B.D., Robinson B.M., Kerr P.G., Pisoni R.L. (2014). High rates of death and hospitalization follow bone fracture among hemodialysis patients. Kidney Int..

[21] Naylor K.L., Garg A.X., Zou G., Langsetmo L., Leslie W.D., Fraser L.A., Adachi J.D., Morin S., Goltzman D., Lentle B. (2015). Comparison of fracture risk prediction among individuals with reduced and normal kidney function. Clin. J. Am. Soc. Nephrol..

[22] Janghorbani M., Feskanich D., Willett W.C., Hu F. (2006). Prospective study of diabetes and risk of hip fracture: The Nurses’ Health Study. Diabetes Care.

[23] Bezerra de Carvalho K.S., Vasco R.F.V., Custodio M.R., Jorgetti V., Moysés R.M.A., Elias R.M. (2019). Chronic kidney disease is associated with low BMD at the hip but not at the spine. Osteoporos. Int..

[24] Hsu C.Y., Chen L.R., Chen K.H. (2020). Osteoporosis in Patients with Chronic Kidney Diseases: A Systemic Review. Int. J. Mol. Sci..

[25] Liu L.F., Shen W.J., Zhang Z.H., Wang L.J., Kraemer F.B. (2010). Adipocytes decrease Runx2 expression in osteoblastic cells: Roles of PPARγ and adiponectin. J. Cell Physiol..

[26] Wang D., Haile A., Jones L.C. (2011). Rosiglitazone-induced adipogenesis in a bone marrow mesenchymal stem cell line—Biomed 2011. Biomed. Sci. Instrum..

